# Supplementary material for: MRE11 liberates cGAS from nucleosome sequestration during tumorigenesis
Source: Nature. 2024 Jan 10;625(7995):585–92. doi: 10.1038/s41586-023-06889-6 (PMC10794148; doi:10.1038/s41586-023-06889-6)
Supplement: Supplementary file 1 — This file contains Supplementary Fig. 1, Supplementary Table 1 and legend to Supplementary Video 1. [file 41586_2023_6889_MOESM1_ESM.pdf]

nature portfolio
https://doi.org/10.1038/s41586-023-06889-6

Supplementary information

MRE11 liberates cGAS from nucleosome sequestration during tumorigenesis

In the format provided by the authors and unedited

SUPPLEMENTAY INFORMATION GUIDE

Mre11 liberates cGAS from nucleosome sequestration during tumorigenesis

Min-Guk Cho, Rashmi J. Kumar, Chien-Chu Lin, Joshua A. Boyer, Jamshaid A. Shahir, Katerina Fagan-Solis, Dennis A. Simpson, Cheng Fan, Christine E. Foster, Anna M. Goddard, Lynn M. Lerner, Simon W. Ellington, Qinhong Wang, Ying Wang, Alice Y. Ho, Pengda Liu, Charles M. Perou, Qi Zhang, Robert K. McGinty, Jeremy E. Purvis, Gaorav P. Gupta

Corresponding authors: garoav_gupta@med.unc.edu

Supplementary figure 1. Raw, unprocessed images of western blots reported in this study

Supplementary table 1. Key resources table

Supplementary video 1. Live-cell imaging of cGAS recruitment to cytoplasmic dsDNA in WT and Mre11-mutant cells

Source Data for Figures 1-4 and Extended Data Figures 2, 3 and 5-10

Supplementary Videos, Source Data and Extended DATA are provided as separate files.

Supplementary Figure 1

Figure 2j

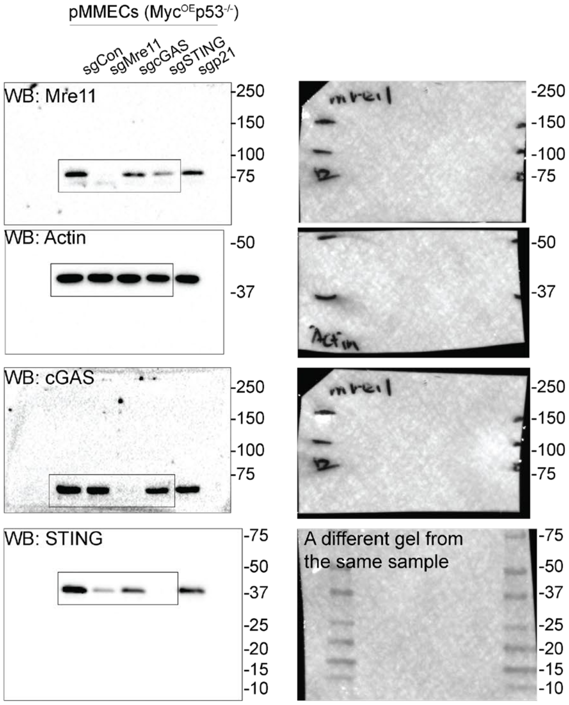

Figure 3j

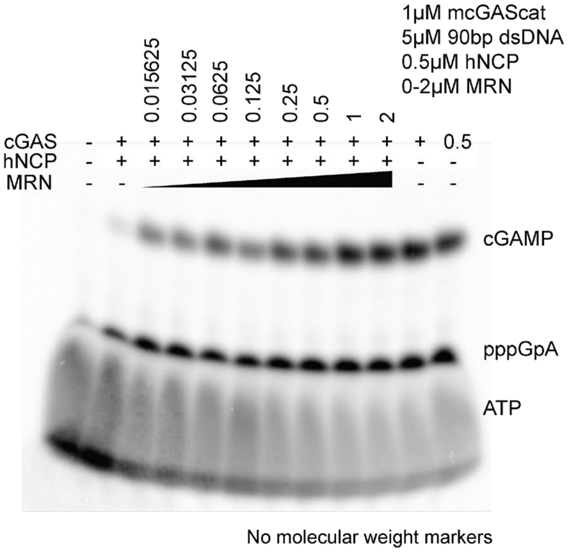

Figure 3d

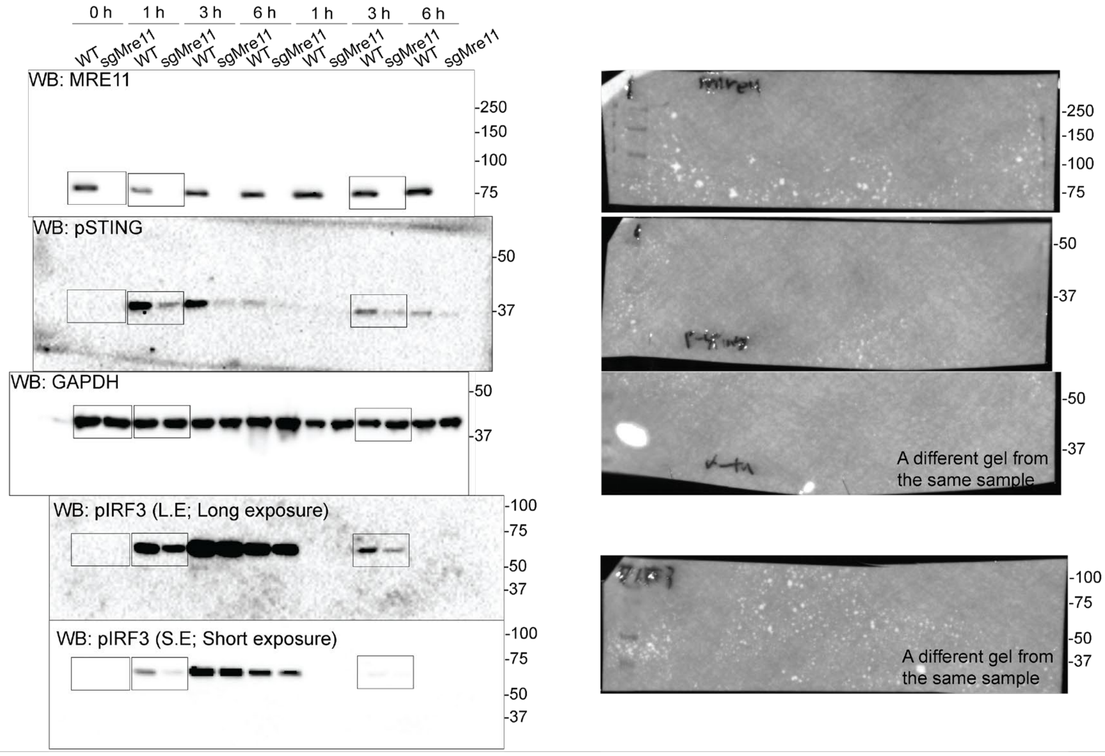

Supplementary Figure 1

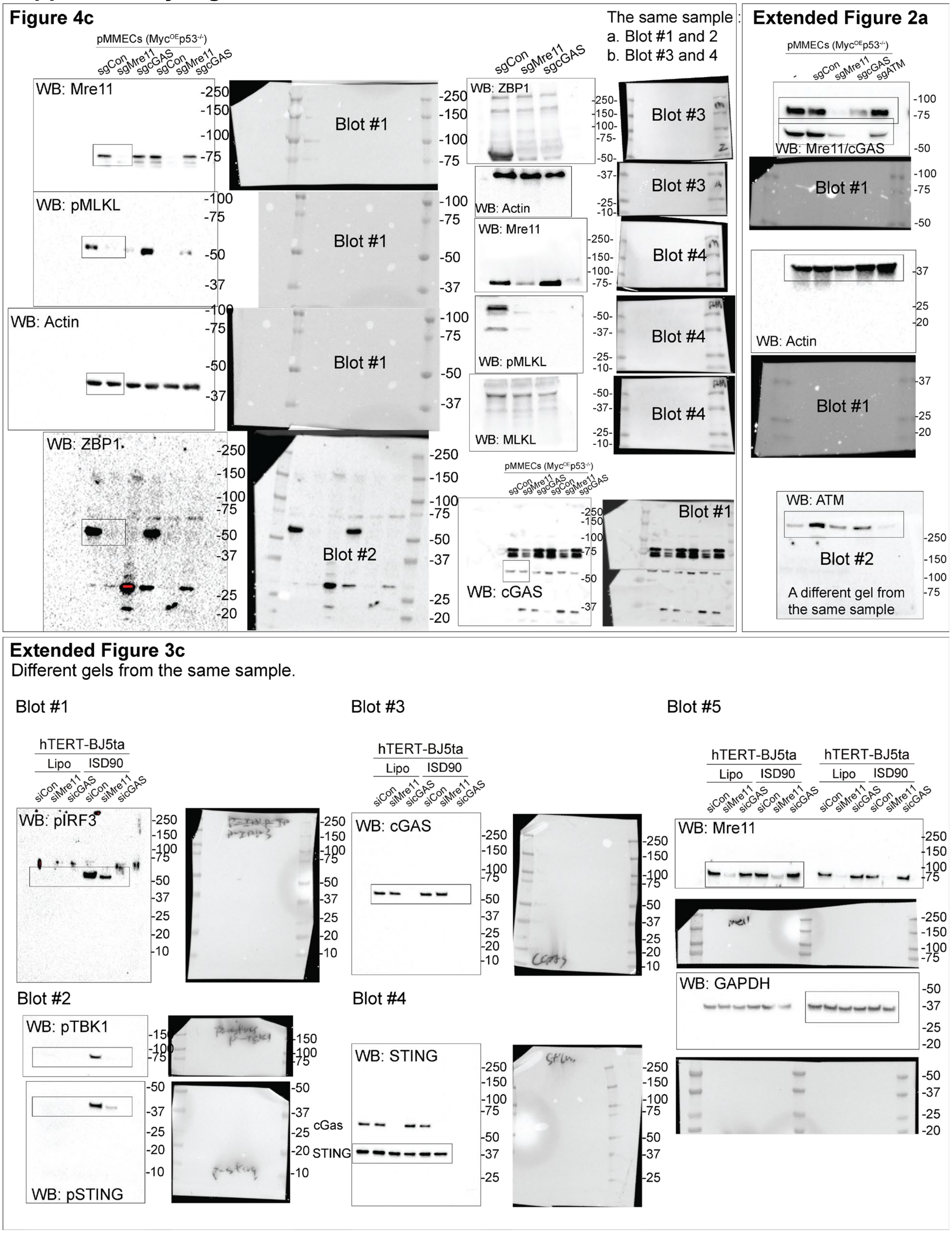

Supplementary Figure 1

Extended Figure 6a

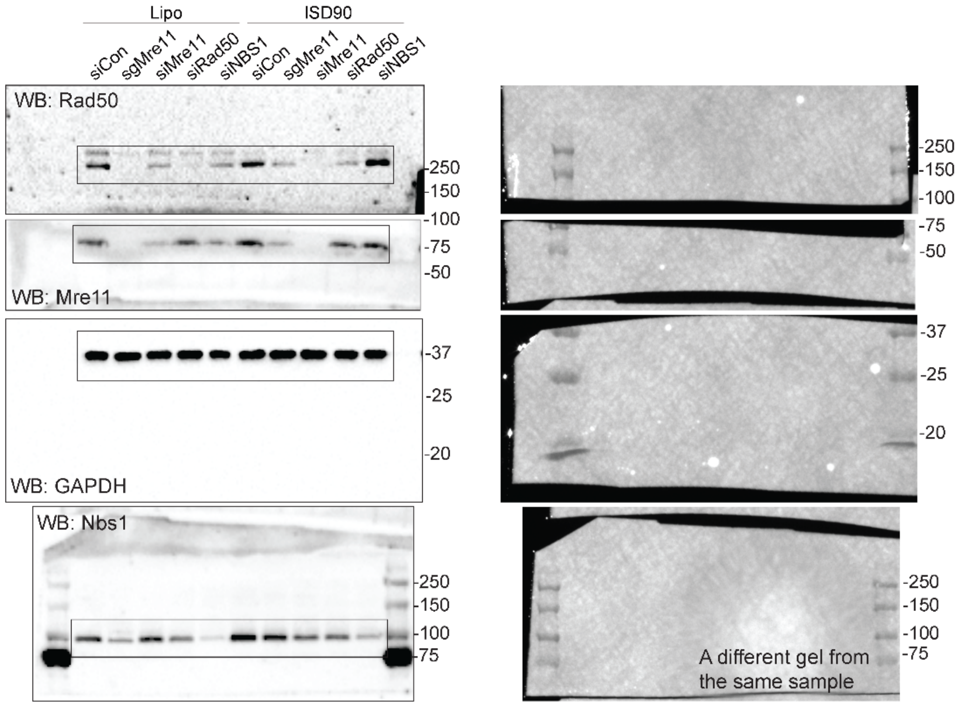

Extended Figure 6d

Different gels from the same sample.

Blot #1

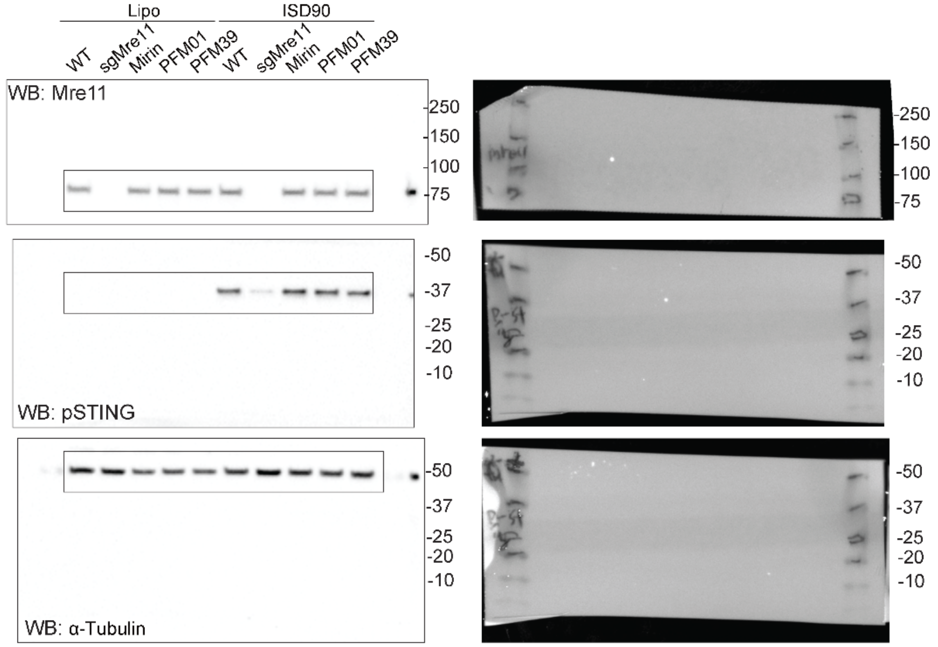

Supplementary Figure 1

Extended Figure 6d

Different gels from the same sample.

Blot #2

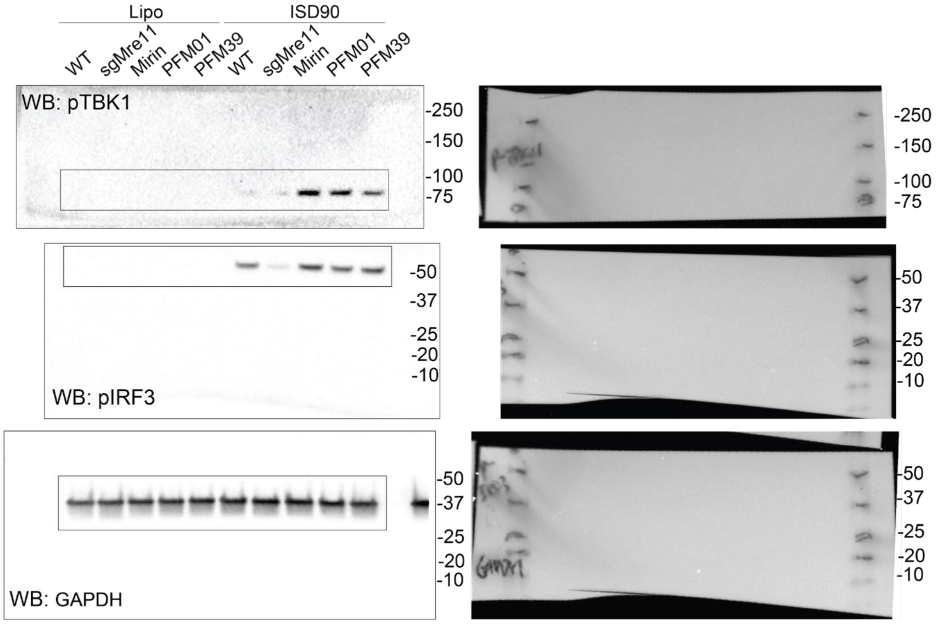

Extended Figure 6f

Different gels from the same sample.

Blot #1

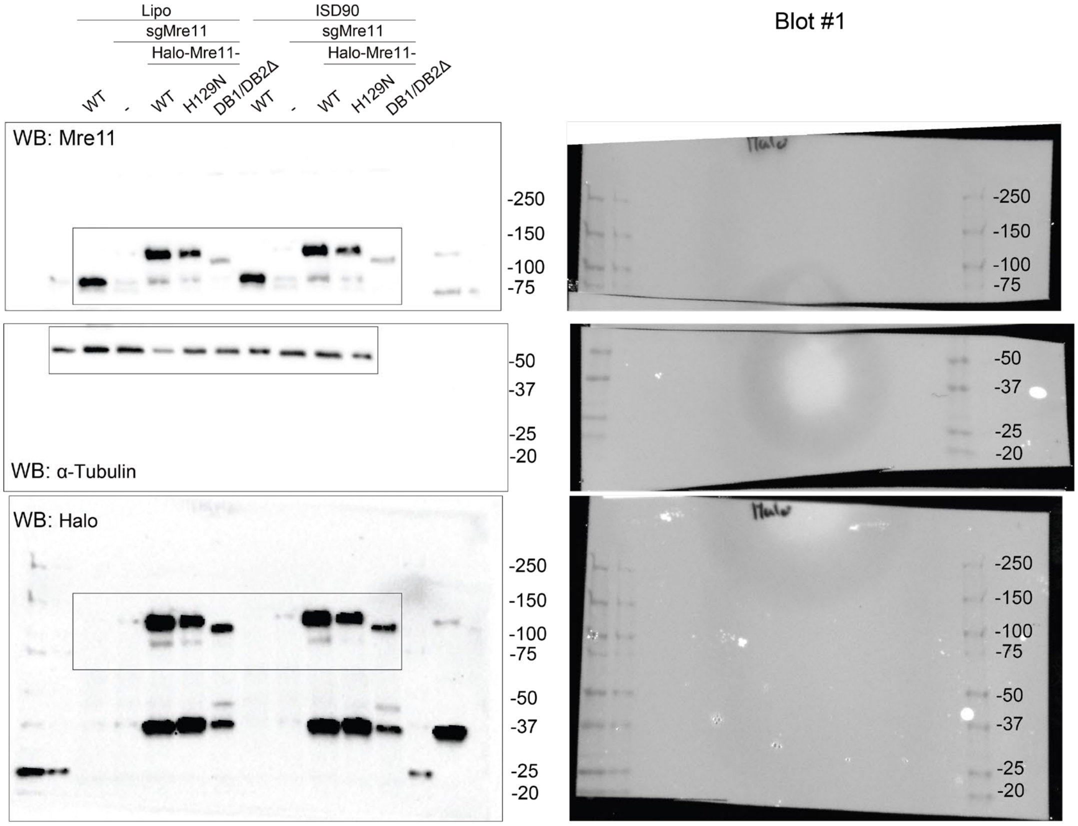

Supplementary Figure 1

Extended Figure 6f

Different gels from the same sample.

Blot #2

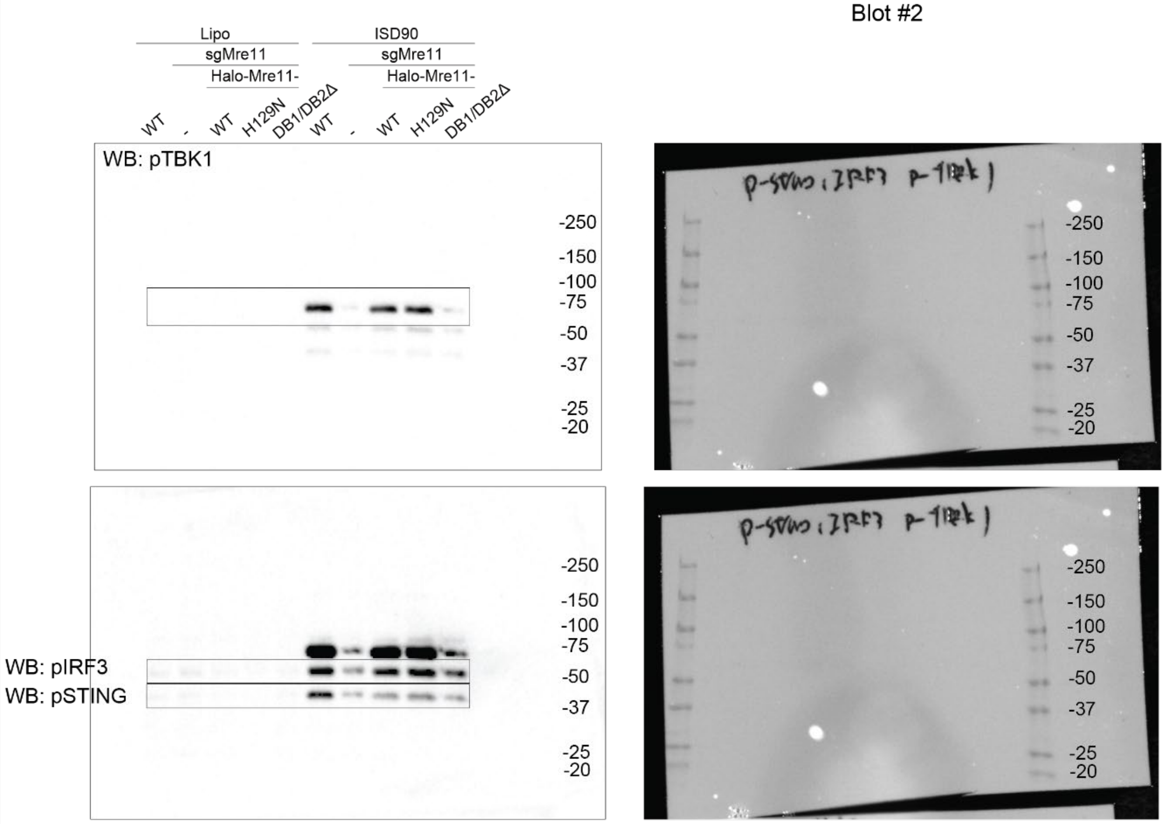

Extended Figure 7d

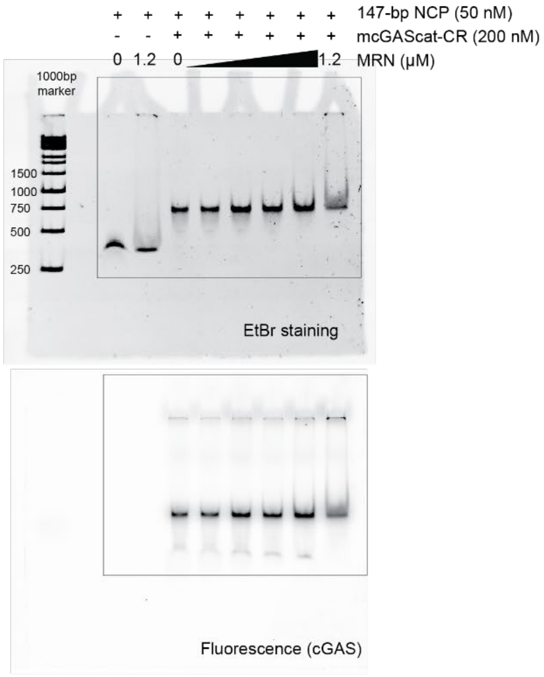

Extended Figure 7e

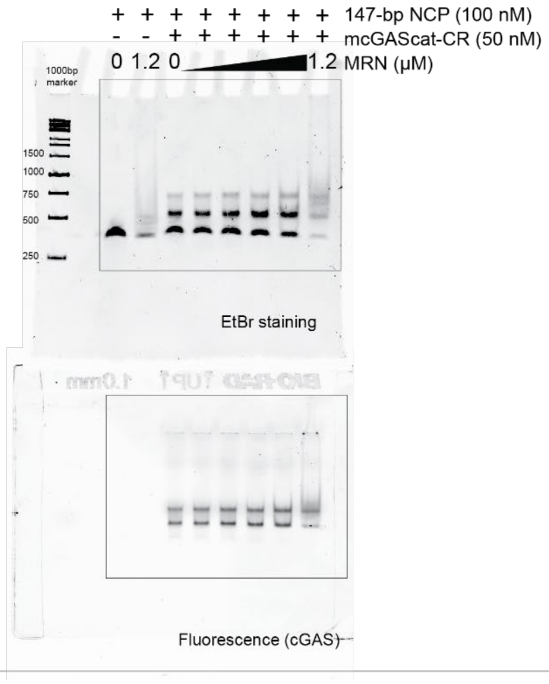

Supplementary Figure 1

Extended Figure 7h

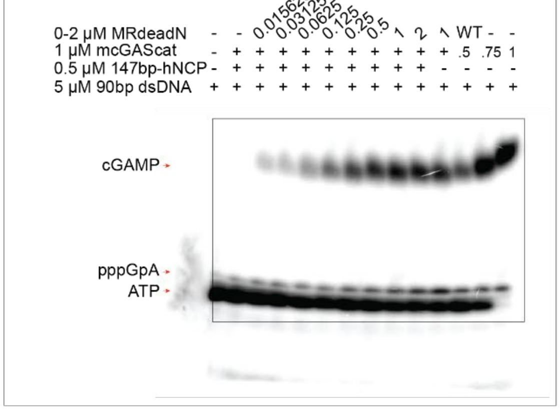

Extended Figure 7i

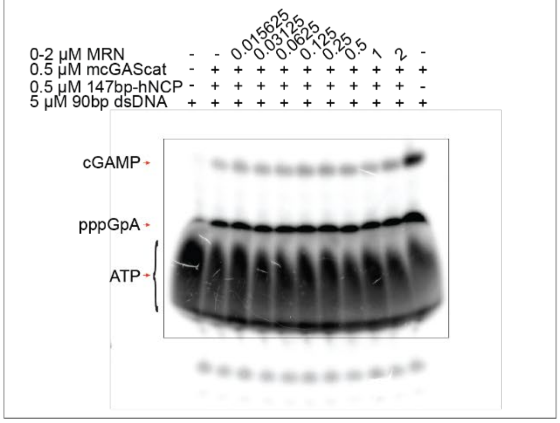

Extended Figure 9c

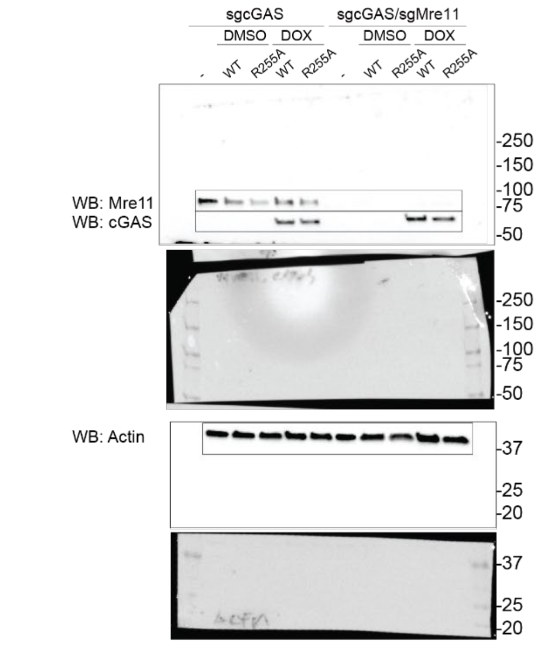

Extended Figure 9a

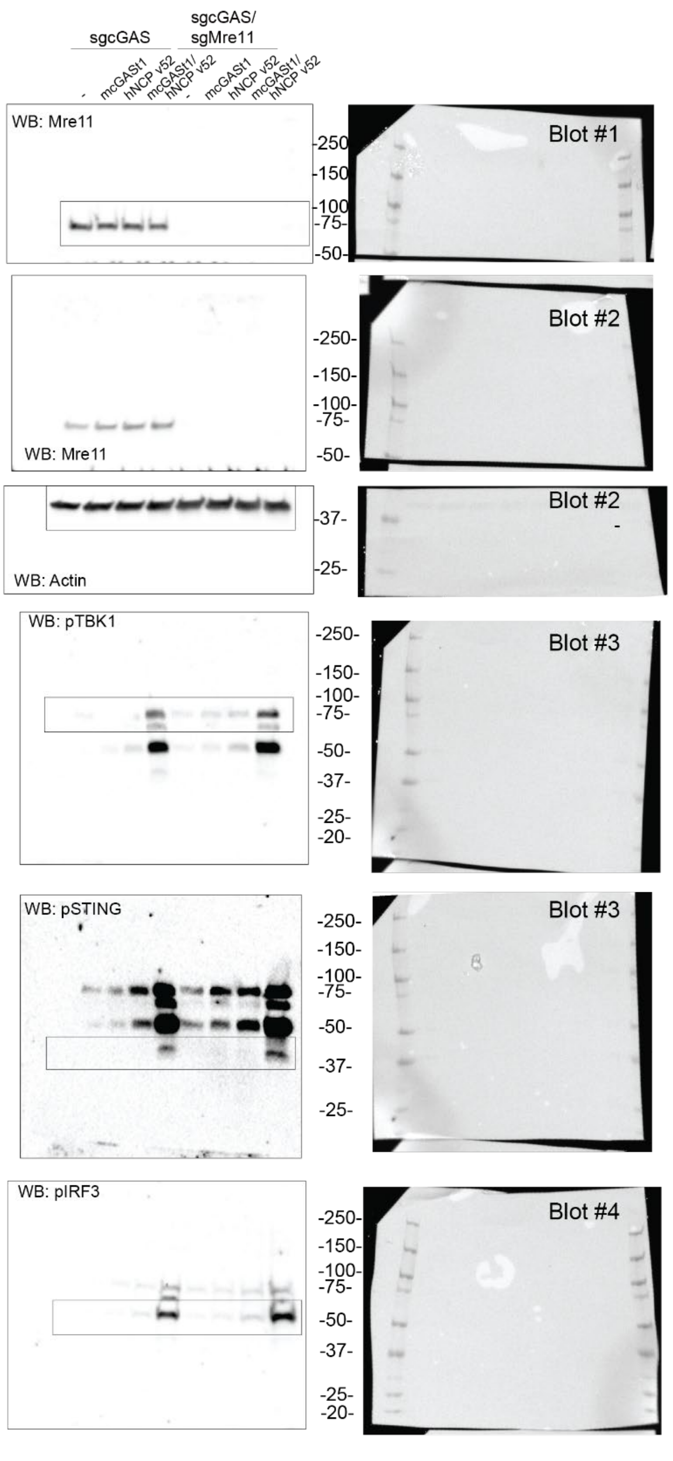

Different gels from the same sample.

Supplementary Figure 1

Extended Figure 10c

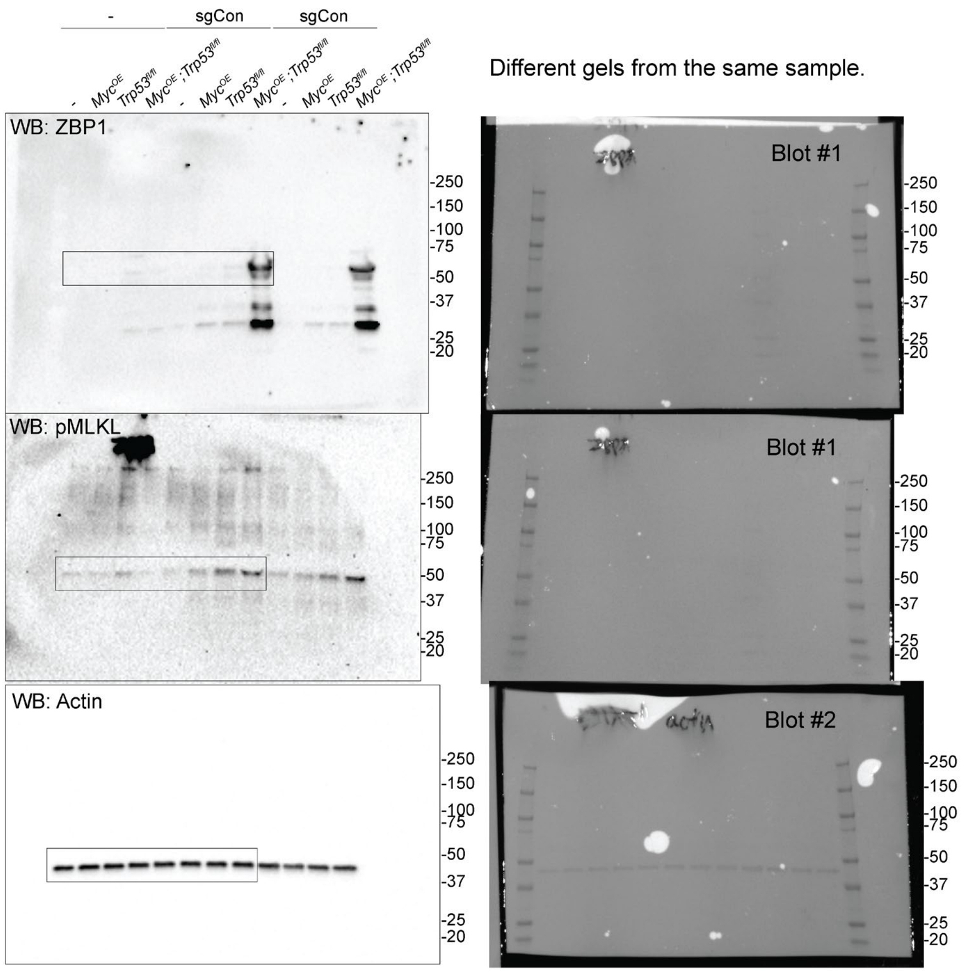

Supplementary Figure 1

Extended Figure 10d

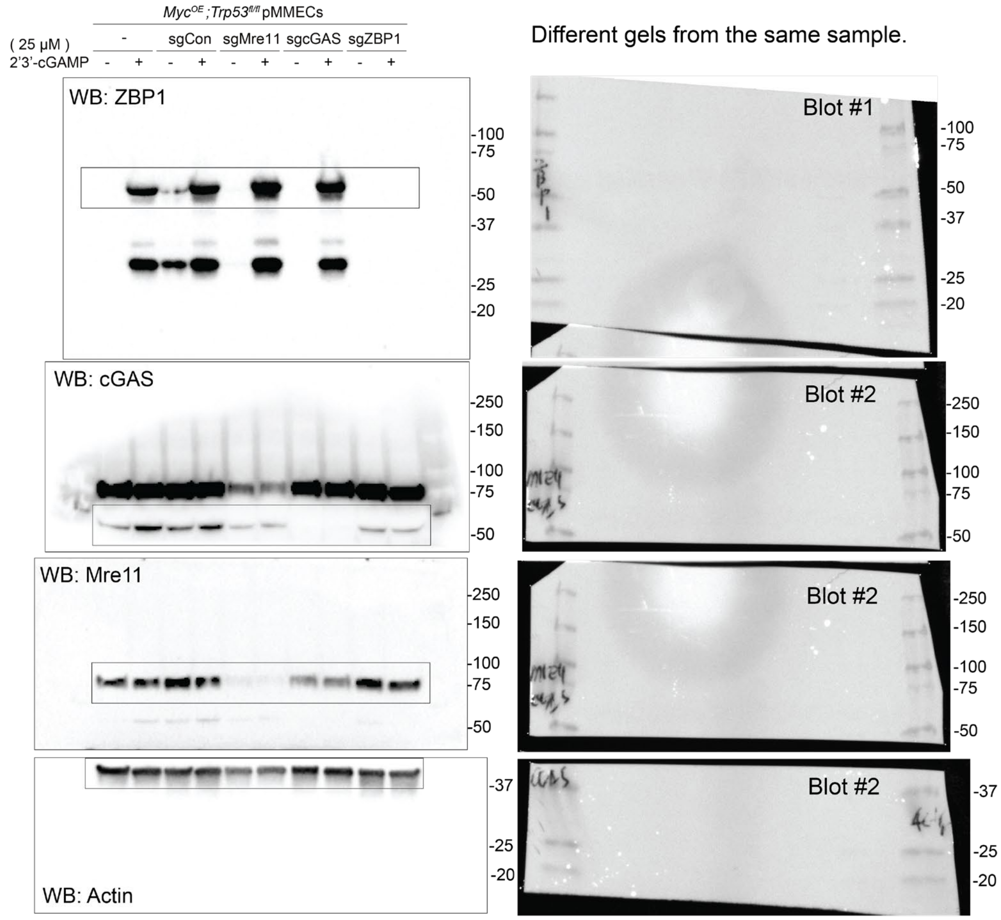

Extended Figure 10f

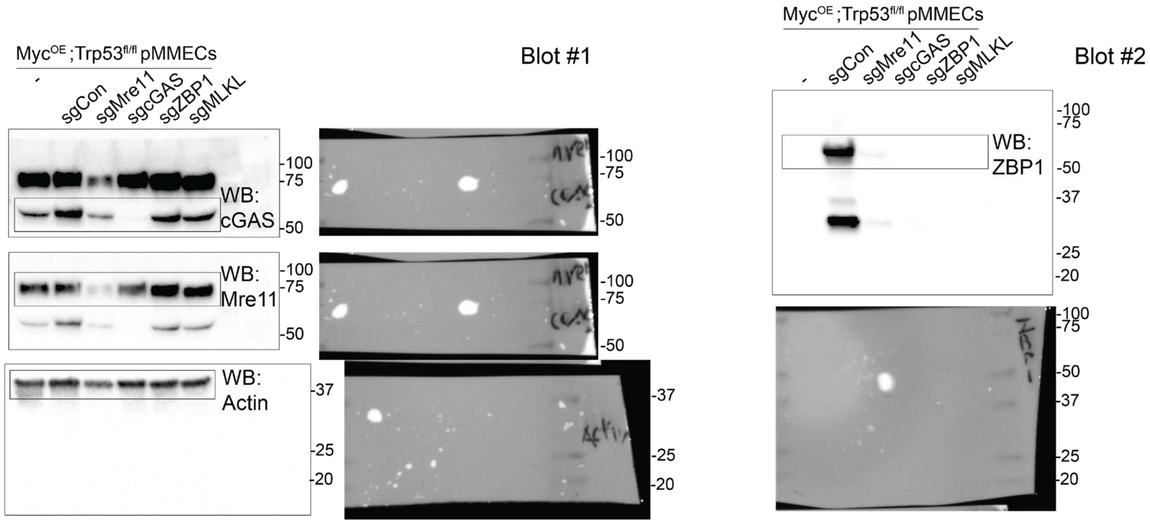

Supplementary Table 1

KEY RESOURCES TABLE

REAGENT or RESOURCE	SOURCE	IDENTIFIER
**Antibodies**		
Horse polyclonal anti-Mouse IgG, HRP-linked antibody (1:5000 for W.B)	Cell Signaling Technology	Cat# 7076S, RRID: AB_330924
Goat polyclonal anti-Rabbit IgG, HRP linked antibody (1:5000 for W.B)	Cell Signaling Technology	Cat# 7074S, RRID: AB_2099233
Goat polyclonal anti-Hamster IgG, HRP-linked antibody (1:5000 for W.B)	Thermo Fisher Scientific	Cat# PA1-29626, RRID: AB_10985385
Rabbit polyclonal anti-Mre11 antibody (1:1000 for W.B)	Novus Biologicals	Cat# NB100-142, RRID: AB_10077796
Mouse monoclonal anti-β-Actin antibody, unconjugated, clone AC-15 (1:5,000 for W.B)	Sigma-Aldrich	Cat# A1978, RRID: AB_476692
Rabbit monoclonal anti-cGAS antibody (D3O8O), Mouse Specific (1:1000 for W.B, 1:500 for ICC)	Cell Signaling Technology	Cat# 31659S, RRID: AB_2799008
Rabbit monoclonal anti-cGAS antibody (D1D3G) (1:1000 for W.B, 1:500 for ICC, 1:4000 for IF)	Cell Signaling Technology	Cat# 15102S, RRID: AB_2732795
Mouse monoclonal anti-α Tubulin antibody (B-7) (1:1000 for W.B, 1:500 for ICC)	Santa Cruz Biotechnology	Cat#SC-5286, RRID: AB_628411
Rabbit monoclonal anti-phospho-Histone H2A.X (Ser139) (20E3), (1:3000 for IF)	Cell Signaling Technology	Cat# 9718S, RRID: AB_2118009
Rabbit polyclonal anti-STING/TMEM173 antibody (1:1000 for W.B)	Novus Biologicals	Cat# NBP2-24683, RRID: AB_2868483
Rabbit monoclonal anti-IRF3 (Ser386) antibody [EPR2346] (1:1000 for W.B)	Abcam	Cat# ab76493, RRID: AB_1523836
Rabbit monoclonal anti-STING (Ser366) antibody (D7C3S) (1:1000 for W.B)	Cell Signaling Technology	Cat# 19781, RRID: AB_2737062
Rabbit monoclonal anti-TBK1/NAK (Ser172) antibody (D52C2) (1:1000 for W.B)	Cell Signaling Technology	Cat# 5483, RRID: AB_10693472
Mouse monoclonal anti-GAPDH antibody (G-9) (1:1000 for W.B)	Santa Cruz Biotechnology	Cat# sc-365062, RRID: AB_10847862
Rabbit polyclonal anti-Rad50 antibody (1:1000 for W.B)	Novus Biologicals	Cat# NBP2-20054, RRID: AB_2894913
Mouse monoclonal anti-NBS1 antibody (1:1000 for WB)	Novus Biologicals	Cat# NB100-221, RRID: AB_10001212
Horse polyclonal anti-Mouse IgG, HRP-linked antibody (1:5000)	Cell Signaling Technology	Cat# 7076S, RRID: AB_330924
Goat polyclonal anti-Rabbit IgG, HRP linked antibody (1:5000)	Cell Signaling Technology	Cat# 7074S, RRID: AB_2099233
Goat polyclonal anti-Hamster IgG (H+L) secondary antibody, HRP (1:5000)	Thermo Fisher Scientific	Cat# PA1-29626, RRID: AB_10985385
Mouse monoclonal anti-HaloTag® protein antibody (1:1000 for WB, 1:500 for I.C.C)	Promega	Cat# G9211, RRID: AB_2688011
Rabbit monoclonal anti-MLKL (phospho S345) [EPR9515(2)] antibody (1:500 for ICC, 1:1000 for WB)	Abcam	Cat# ab196436, RRID: AB_2687465
Mouse monoclonal anti-MLKL antibody (1:1000 for WB)	Proteintech	Cat# 66675-1-Ig, RRID: AB_2882029
Mouse monoclonal anti-ZBP1 (Zippy-1) antibody (1:1000 for WB)	AdipoGen	Cat# AG-20B-0010, RRID: AB_2490191

Rabbit monoclonal anti-ATM (D2E2) (1:1000 for WB)	Cell Signaling Technology	Cat# 2873S RRID: AB_2062659
Goat anti-Rabbit IgG (H+L) Secondary Antibody, Alexa Fluor 488	Thermo Fisher Scientific	Cat# A11034 RRID: AB_2576217
Goat anti Rabbit IgG (H+L) Secondary Antibody, Alexa Fluor 594	Thermo Fisher Scientific	Cat# A11037 RRID: AB_2534095
F(ab')2-Goat anti-Rabbit IgG (H+L) Cross-Adsorbed Secondary Antibody, Alexa Fluor 633 (1:500 for ICC)	Thermo Fisher Scientific	Cat# A21072 RRID: AB_1500777
Cy™3 AffiniPure Donkey Anti-Mouse IgG (H+L) (1:500 for ICC)	Jackson ImmunoResearch	Cat# 715-165-151 RRID: AB_2315777
Cy™5 AffiniPure Goat Anti-Rabbit IgG (H+L) (1:500 for ICC)	Jackson ImmunoResearch	Cat# 111-175-144 RRID: AB_2338013
Streptavidin, Alexa Fluor™ 488 Conjugate (1:500 for ICC)	Thermo Fisher Scientific	Cat# S32354, RRID: AB_2315383
Sheep Polyclonal anti-Z-DNA (1:500 for ICC)	Novous Biologicals	Cat# NB100-749 RRID: AB_10003363
Donkey anti-Sheep IgG (H+L) Cross-Adsorbed Secondary Antibody, Alexa Fluor™ 594 Invitrogen™ (1:500 for ICC)	Invitrogen	Cat# A-11016 RRID: AB_2534083
**Bacterial and Virus Strains**		
Endura DUOs Electrocompetent Cells	Lucigen	60242-2
Endura Competent Cells	Lucigen	60241-2
Invitrogen™ MAX Efficiency™ DH10Bac Competent Cells	Thermo Fisher Scientific	10361012
One Shot® ccdB Survival™ 2 T1R Competent cells	Invitrogen	A10460
Subcloning Efficiency™ DH5α Competent Cells	Thermo fisher scientific	18265017
**Chemicals, Peptides, and Recombinant Proteins**		
Liberase Blendzyme 2	Roche	11988425001
EpiCult-B Mouse Medium kit	Stem Cell Technologies	05610
DNase I	Worthington	LS002060
Dispase (5 U/mL)	Stem Cell Technologies	07913
LA7 medium	Thermo Fisher Scientific	11965084
Insulin	VWR	11061-68-0
150 U/mL Collagenase type 3	Worthington	LS004182
Cultrex 3D Culture Matrix Rat Collagen I	Fisher Scientific	344702001
Trypsin EDTA	Gibco	25200-056
Bovine Serum Albumin	Fisher Scientific	BP9706-160
Carbenicillin	Fisher Scientific	BP26481
Ampicillin	Fisher Scientific	B1760-25
4′,6-Diamidine-2′-phenylindole dihydrochloride (DAPI)	Sigma-Aldrich	10236276001; CAS: 28718-90-3
C-176 (STING inhibitor) 10mM/1mL	Selleck Chemicals	S6575; CAS: 1032350-13-2
2’3’-cGAMP, 1 mg	Invivogen	tlrl-nacga23-1
Necrosulfonamide (NSA; MLKL inhibitor)	Selleck Chemicals	S8251; CAS: 1360614-48-7
HS-1371 (RIPK3 inhibitor)	Selleck Chemicals	S8775; CAS: 2158197-70-5
Polyethylenimine, Linear (MW 25,000)	Polysciences	23966-2

Hexadimethrine bromide/polybrene	Sigma-Aldrich	107689; CAS: 28728-55-4
Janelia Fluor® 646 HaloTag® Ligands	Promega	GA1120
Bond Dewax Solution	Leica	AR9222
Bond Wash Solution	Leica	AR9590
Bond-epitope retrieval solution 2 pH9.0	Leica	AR9640
Novolink Polymer	Leica	RE7161
TSA Cyanine 5 (Cy5)	Akoya Biosciences	FP1117
TSA Cyanine 3 (Cy3)	Akoya Biosciences	FP1046
Bond-epitope retrieval solution 1 pH 6.0	Leica	AR9961
2x Laemmli sample buffer	Bio-Rad	161-0737
Sf-900™ III SFM	Thermo Fisher Scientific	12658019
cOmplete EDTA-free protease inhibitor cocktail	Sigma-Aldrich	11873580001
3X FLAG® Peptide	Sigma-Aldrich	F4799
Hoechst 33258	Invitrogen	H3569
Mirin	Sigma-Aldrich	M9948
PFM39	Sigma-Aldrich	SML1839
PFM01	Sigma-Aldrich	SML1735
BP Clonase II Enzyme Mix	Thermo Fisher Scientific	11789100
LR Clonase II Enzyme Mix	Thermo Fisher Scientific	11791100
*Dpn*1	NEB	R0176S
Doxycycline hyclate	Sigma-Aldrich	D9891
Normocin™	Invivogen	ant-nr-05
QUANTI-Luc	Invivogen	rep-qlc1
SpCas9 2NLS Nuclease	Synthego	N/A
Purified 3xFLAG® peptide	Sigma-Aldrich	F4799
Inorganic Pyrophosphatase (IPP)	Sigma-Aldrich	I1643
ATP [α-^{32}P]	PerkinElmer	NEG003X250UC
Biotin-maleimide	Sigma-Aldrich	B1267
Atto 647N maleimide	Sigma-Aldrich	05316-1MG-F
LANCE Ultra ULight-anti-6xHis	PerkinElmer	TRF0134-M
**Critical Commercial Assays**		
PlasmoTest	Invitrogen	REP-PT1
RNeasy Plus Mini Kit	Qiagen	74136
Maxima First Strand cDNA Synthesis Kit for RT-qPCR, with dsDNase	Thermo Fisher Scientific	FERK1672
Fast SYBR™ Green Master Mix	Thermo Fisher Scientific	4385617
Q5® Hot Start High-Fidelity 2X Master Mix	NEB	M0494S
NEBuilder® HiFi DNA Assembly Master Mix	NEB	E2621L
HIFI DNA assembly Master Mix	NEB	E2611S
2'3'-cGAMP ELISA Kit	Cayman Chemical Company	Item No. 501700
EdU-Click 594	Sigma-Aldrich	BCK-EDU594
**Experimental Models: Cell Lines**		
HEK 293T/17	ATCC	ATCC® CRL-11268
WT MEFs	Gift from John Petrini, Ph.D	N/A
ATLD/ATLD MEFs	Gift from John Petrini, Ph.D	N/A
BJ-5ta	ATCC	CRL-4001
MDA-MB-231	ATCC	CRM-HTB-26
sgMREe11 MDA-MB-231	In this paper	N/A

sgMRE11 + Halo-hMRE11-WT MDA-MB-231	In this paper	N/A
sgMRE11 + RFP-cGAS MDA-MB-231	In this paper	N/A
sgMRE11 + RFP-cGAS + Halo-hMRE11 MDA-MB-231	In this paper	N/A
sgMre11 + Halo-hMre11-H129N MDA-MB-231	In this paper	N/A
sgMre11 + Halo-hMRE11-DB1△/DB2△ MDA-MB-231	In this paper	N/A
sgcGAS MDA-MB-231	In this paper	N/A
sgMre11 + sgcGAS MDA-MB-231	In this paper	N/A
sgcGAS + cGAS-WT MDA-MB-231	In this paper	N/A
sgcGAS + cGAS-R255A MDA-MB-231	In this paper	N/A
sgMre11 + sgcGAS + cGAS-WT MDA-MB-231	In this paper	N/A
sgMre11 + sgcGAS + cGAS-R255A MDA-MB-231	In this paper	N/A
Lucia^{TM} ISG Cells	Invivogen	rawl-isg
Sf9 cells in Sf-900™ II SFM	Thermo Fisher Scientific	11496015
**Experimental Models: Organisms/Strains**		
FVB.129P2-*Trp53*^{tm1Brn}/Nci (*Mus musculus*) Referred to in this manuscript as "*Trp53*^{FL}"	Frederick National Laboratory for Cancer Research	01XC2
C57BL/6N-*Gt(ROSA)26Sor*$^{tm13(CAG-MYC,-CD2^{*})Rsky}$/J (*Mus musculus*) Referred to in this manuscript as "*R26*^{MycOE}"	The Jackson Laboratory	020458
B6;129-*Gt(ROSA)26Sor*$^{tm1(CAG-cas9^{*},-EGFP)Fezh}$/J (*Mus musculus*) Referred to in this manuscript as "*R26*^{Cas9}"	The Jackson Laboratory	024857
NOD.129S7(B6)-*Rag1*^{tm1Mom}/J (*Mus musculus*) Referred to in this manuscript as "*NRG*"	The Jackson Laboratory	003729
**Oligonucleotides**		
Primer: Mouse IFIT1<br>Forward: TACAGGCTGGAGTGTGCTGAGA<br>Reverse: CTCCACTTTCAGAGCCTTCGCA	Eton Bioscience	N/A
Primer: Mouse IFNB1<br>Forward: GCCTTTGCCATCCAAGAGATGC<br>Reverse: ACACTGTCTGCTGGTGGAGTTC	Eton Bioscience	N/A
Primer: Mouse CCL5<br>Forward: CCTGCTGCTTTGCCTACCTCTC<br>Reverse: ACACACTTGGCGGTTCCTTCGA	Eton Bioscience	N/A
Primer: Mouse ZBP1<br>Forward: GATCTACCACTCACGTCAGGAAG<br>Reverse: GGCAATGGAGATGTGGCTGTTG	Eton Bioscience	N/A
Primer: Human CCL5<br>Forward: CCTGCTGCTTTGCCTACATTGC<br>Reverse: ACACACTTGGCGGTTCTTTCGG	Eton Bioscience	N/A
Primer: Human CXCL10<br>Forward: GGTGAGAAGAGATGTCTGAATCC<br>Reverse: GTCCATCCTTGGAAGCACTGCA	Eton Bioscience	N/A
Primer: Human IFIT1<br>Forward: GCCTTGCTGAAGTGTGGAGGAA<br>Reverse: ATCCAGGCGATAGGCAGAGATC	Eton Bioscience	N/A
siGENOME Human MRE11 (4361) siRNA - SMARTpool	Horizon Discovery	M-009271-01
ON-TARGETplus Human RAD50 (10111) siRNA - SMARTpool	Horizon Discovery	L-005232-00

ON-TARGETplus Human NBN (4683) siRNA - SMARTpool	Horizon Discovery	L-009641-00
ON-TARGETplus Non-targeting Pool	Horizon Discovery	D-001810-10
dsDNA : ISD90<br>Forward :<br>TACAGATCTACTAGTGATCTATGACTGATC<br>TGTACATGATCTACATACAGATCTACTAGT<br>GATCTATGACTGATCTGTACATGATCTACA<br>Reverse :<br>TGTAGATCATGTACAGATCAGTCATAGATC<br>ACTAGTAGATCTGTATGTAGATCATGTACA<br>GATCAGTCATAGATCACTAGTAGATCTGTA	Integrated DNA Technologies	N/A
dsDNA : Biotin-ISD90<br>Forward :<br>/5BiosG/TACAGATCTACTAGTGATCTATGAC<br>TGATCTGTACATGATCTACATACAGATCTA<br>CTAGTGATCTATGACTGATCTGTACATGAT<br>CTACA	Integrated DNA Technologies	N/A
dsDNA : Alexa488-ISD90<br>Forward :<br>/5Alexa488/TACAGATCTACTAGTGATCTATG<br>ACTGATCTGTACATGATCTACATACAGATC<br>TACTAGTGATCTATGACTGATCTGTACATG<br>ATCTACA<br>Reverse :<br>TGTAGATCATGTACAGATCAGTCATAGATC<br>ACTAGTAGATCTGTATGTAGATCATGTACA<br>GATCAGTCATAGATCACTAGTAGATCTGTA	Integrated DNA Technologies	N/A
dsDNA : Alexa594-ISD90<br>Forward :<br>/5Alexa594/TACAGATCTACTAGTGATCTATG<br>ACTGATCTGTACATGATCTACATACAGATC<br>TACTAGTGATCTATGACTGATCTGTACATG<br>ATCTACA<br>Reverse :<br>TGTAGATCATGTACAGATCAGTCATAGATC<br>ACTAGTAGATCTGTATGTAGATCATGTACA<br>GATCAGTCATAGATCACTAGTAGATCTGTA	Integrated DNA Technologies	N/A
Alt-R® CRISPR-Cas9 crRNA for human MRE11<br>GCCGATGGTGAAGTGGTAAG	Integrated DNA Technologies	N/A
CRISPR gene knockout Kit V2 (human MB21D1)	Synthego	N/A
sgCon sequence for Gibson cloning<br>CTGATTTGAATAATGATGCC	Eton Bioscience	N/A
sgMRE11 sequence for Gibson cloning<br>TGGAGATCACTACTCGAGGC	Eton Bioscience	N/A
sgcGAS sequence for Gibson cloning<br>AAACGGCTCTCGTCTTAGAT	Eton Bioscience	N/A
sgSTING sequence for Gibson cloning<br>CGGCAGTTATTTCGAGACTC	Eton Bioscience	N/A
sgZBP1 #1 sequence for Gibson cloning<br>CAGGTGTTGAGCGATGACGG	Eton Bioscience	N/A
sgZBP1 #2 sequence for Gibson cloning<br>ACCTCTTCCTTCACCTCGCG	Eton Bioscience	N/A

sgZBP1 #3 sequence for Gibson cloning ACGGCGGCCCTGTGAAGAT	Eton Bioscience	N/A
sgRIPK3 sequence for Gibson cloning CCCGGACACGAAGTCCCAC	Eton Bioscience	N/A
sgMLKL sequence for Gibson cloning CACACGGTTTCCTAGACGC	Eton Bioscience	N/A
sgATM sequence for Gibson cloning AGACCTCTTGAATTATGTCA	Eton Bioscience	N/A
Primer for attB-cGAS-WT-attBForward : GGGGACAAGTTTGTACAAAAAAGCAGGCTT CATGCAGCCTTGGCACGGAAAG Reverse : GGGGACCACTTTGTACAAGAAAGCTGGGT TTCAAAATTCATCAAAAACTGG	Integrated DNA Technologies	N/A
Primer for site-Directed mutagenesis (cGAS-R255 -> A) Forward : ATTTAAAGCAAATCCGAAAGAAAATCCTCT GA Reverse : GGATTTGCTTTAAATTTCACAAAGTAATATG CA	Integrated DNA Technologies	N/A
gBlocks™ Gene Fragments (BstBI-MRE11-H129N-BstBI)	Integrated DNA Technologies	N/A
gBlocks™ Gene Fragments (BstBI-MRE11-DB1∆/DB2∆-BstBI)	Integrated DNA Technologies	N/A
**Recombinant DNA**		
pLV-Cre_LKO1	Addgene	25997
LentiCRISPR-Cre-V2-LumiFluor	Addgene	52961
psPAX2	Addgene	12260
pMD2.G	Addgene	12259
LentiCRISPR-Cre-V2-sgControl-LumiFluor	This Paper	N/A
LentiCRISPR-Cre-V2-sgMRE11-LumiFluor	This Paper	N/A
LentiCRISPR-Cre-V2-sgcGAS-LumiFluor	This Paper	N/A
LentiCRISPR-Cre-V2-sgSTING-LumiFluor	This Paper	N/A
LentiCRISPR-Cre-V2-sgZBP1-LumiFluor	This Paper	N/A
LentiCRISPR-Cre-V2-sgRIPK3-LumiFluor	This Paper	N/A
LentiCRISPR-Cre-V2-sgMLKL-LumiFluor	This Paper	N/A
LentiCRISPR-Cre-V2-sgATM-LumiFluor	This Paper	N/A
Lentiviral_pRRL-EF1a-GpNLuc	Gift from Antonio Amelio, Ph.D	N/A
pBABE-Puro-Halo-MRE11-WT	Gift from Eli Rothenberg at NYU	N/A
pBABE-Puro-Halo	Gift from Eli Rothenberg at NYU	N/A
pBABE-Puro-Halo-MRE11-H129N	This Paper	N/A
pBABE-Puro-Halo-MRE11-DB1∆/DB2∆	This Paper	N/A
pTRIP-CMV-tagRFP-FLAG-cGAS	Addgene	#86676
pTRIP-CMV-GFP-FLAG-cGAS	Addgene	#86675
pUMVC	Addgene	#8449
pCMV-VSV-G	Addgene	#8454
pLV-PCNA-mCherry	From Jeremy Purvis at UNC	N/A
Human MRE11-FLAG (pTP813)	Addgene	#113308
RAD50-6xHIS (pTP2620)	Addgene	#113311
NBS1-FLAG (pTP288)	Addgene	#113460

pET-6xHis-Sumo-mcGAS	From Pingwei Li @ Texas A&M	N/A
pET-6xHis-Sumo-hcGAS	From Pingwei Li @ Texas A&M	N/A
pBig1a-hRAD50-hMRE11N2xSTR	This paper	N/A
pBig1a-hRAD50-hMRE11(H129N)N2xSTR	This paper	N/A
pDONR^{TM}221	Thermo Fisher Scientific	12536017
PB-TA-ERP2	Addgene	#80477
PB-TA-ERP2-hcGAS-WT	This paper	N/A
PB-TA-ERP2-hcGAS-R255A	This paper	N/A
Super PiggyBac Transposase Expression Vector	System Biosciences	PB210PA-1
**Software and Algorithms**		
BowTie2 v2.3.4.1	Langmead, B. and S. L. Salzberg (2012). "Fast gapped-read alignment with Bowtie 2." Nat Methods **9**(4): 357-359.	https://sourceforge.net/projects/bowtie-bio/files/bowtie2/2.3.4.1
Samtools v1.6.0	Li, H., B. Handsaker, A. Wysoker, T. Fennell, J. Ruan, N. Homer, G. Marth, G. Abecasis, R. Durbin and S. Genome Project Data Processing (2009). "The Sequence Alignment/Map format and SAMtools." Bioinformatics **25**(16): 2078-2079	http://www.htslib.org/download/
BedTools v2.26.0	Quinlan, A. R. and I. M. Hall (2010). "BEDTools: a flexible suite of utilities for comparing genomic features." Bioinformatics **26**(6): 841-842.	https://github.com/arq5x/bedtools2
Python ≥v3.5	G. van Rossum, Python tutorial, Technical Report CS-R9526, Centrum voor Wiskunde *en* Informatica *(CWI),* Amsterdam, *May* 1995	https://www.python.org/
Ginkgo	Garvin, T., R. Aboukhalil, J. Kendall, T. Baslan, G. S. Atwal, J. Hicks, M. Wigler and M. C. Schatz (2015). "Interactive analysis and assessment of single-cell copy-number variations." Nat Methods **12**(11): 1058-1060.	http://qb.cshl.edu/ginkgo/?q=
Vџlur algorithm	This paper	GitHub
GNU Gzip v1.5	N.A.	https://www.gnu.org/software/gzip/
Graphpad Prism v7, v8 and v9	GraphPad Prism Inc	https://www.graphpad.com/
Python-Levenshtein Library v0.12.0	N.A.	https://github.com/ztane/python-Levenshtein

BioEdit Sequence Alignment Editor	Hall, T.A. 1999. BioEdit: a user-friendly biological sequence alignment editor and analysis program for Windows 95/98/NT. Nucl. Acids. Symp. Ser. 41:95-98.	http://www.mbio.ncsu.edu/BioEdit/bioedit.html
Fiji ImageJ 1.53c<br>Schneider et al. 2012<br>https://imagej.nih.gov/ij	Schindelin, J.; Arganda-Carreras, I. & Frise, E. et al. (2012), "*Fiji: an open-source platform for biological-image analysis*", Nature methods 9(7): 676-682, *PMID 22743772*, doi:*10.1038/nmeth.2019 (on Google Scholar).*	https://imagej.net/Fiji#Downloads
SnapGene software v4.3.4	GSL Biotech	https://www.snapgene.com
ZEN 2011 microscope software	ZEISS	https://www.Zeiss.com/corporate/int/home.html
ImageJ (v. 2.0.0-rc-43/1.51k)	NIH	https://imagej.nih.gov/ij/index.html
NIS Elements AR software v.4.50	Nikon	https://www.nikon.com/products/microscope-solutions/lineup/img_soft/nis-elements/
NIS-Elements Viewer v.4.2	Nikon	https://www.nikon.com/products/microscope-solutions/lineup/img_soft/nis-elements/
**Other**		
Mouse Reference Sequence GRCm38	www.Ensembl.org	https://www.ensembl.org/Mus_musculus/Info/Index
Microsatellites, CpG Islands, Simple Repeats, SINE Elements, LINE Elements, LTR	UCSC Genome Browser	https://genome.ucsc.edu/cgi-bin/hgTables
Genes	www.Ensembl.org	Ensembl v91
Oregon-green hNCP	This paper	N/A
hNCP	This paper	N/A
Trans-Blot	Bio-Rad	1704150
Qubit 3.0 Fluorometer	Thermo Fisher Scientific	Q33216
RS2000 X-irradiator	Rad Source	020712
Luminometer	SpectraMax i3x	N/A
Neon™ Transfection System	Invitrogen	MPK5000
Neon™ Transfection System 10 μL Kit	Invitrogen	MPK1025
Typhoon FLA-9500 imager	GE Healthcare	N/A
ANTI-FLAG® M2 Affinity Gel	Sigma-Aldrich	A2220
Matrigel® Matrix	Corning	356231
Source Q resin	GE healthcare	17094705
Superose 6 Increase 10/300 GL; Polyethersulfone/Pore size : 50,000 MWCO	Vivascience	29-0915-96
Zeba Spin desalting column	Thermo Fisher Scientific	89878

5-(and-6-)-Carboxyrhodamine 6G, succinimidyl ester	Thermo Fisher Scientific	C6157
Strep-tactin XT	IBA Lifesciences	2-4030-010
Superdex 200 increase columns	GE healthcare	28990944
Talon resin	Clontech	635504
Source S resin	Ge healthcare	17094405

CONTACT FOR REAGENT AND RESOURCE SHARING

Further information and requests for resources and reagents should be directed to and will be fulfilled by the Lead Contact, Dr. Gaorav Gupta: gaorav_gupta@med.unc.edu.
