# Supplementary material for: MRE11 liberates cGAS from nucleosome sequestration during tumorigenesis
Source: Nature. 2024 Jan 10;625(7995):585–92. doi: 10.1038/s41586-023-06889-6 (PMC10794148; doi:10.1038/s41586-023-06889-6)
Supplement: Supplementary file 2 — Reporting Summary [file 41586_2023_6889_MOESM2_ESM.pdf]

nature portfolio

Corresponding author(s): Gaorav P. Gupta, MD PhD, gaorav@med.unc.edu

Last updated by author(s): Aug 28, 2023

Reporting Summary

Nature Portfolio wishes to improve the reproducibility of the work that we publish. This form provides structure for consistency and transparency in reporting. For further information on Nature Portfolio policies, see our Editorial Policies and the Editorial Policy Checklist.

Statistics

For all statistical analyses, confirm that the following items are present in the figure legend, table legend, main text, or Methods section.

n/a	Confirmed	
☐	☒	The exact sample size (*n*) for each experimental group/condition, given as a discrete number and unit of measurement
☐	☒	A statement on whether measurements were taken from distinct samples or whether the same sample was measured repeatedly
☐	☒	The statistical test(s) used AND whether they are one- or two-sided<br>*Only common tests should be described solely by name; describe more complex techniques in the Methods section.*
☒	☐	A description of all covariates tested
☐	☒	A description of any assumptions or corrections, such as tests of normality and adjustment for multiple comparisons
☐	☒	A full description of the statistical parameters including central tendency (e.g. means) or other basic estimates (e.g. regression coefficient) AND variation (e.g. standard deviation) or associated estimates of uncertainty (e.g. confidence intervals)
☐	☒	For null hypothesis testing, the test statistic (e.g. F, t, r) with confidence intervals, effect sizes, degrees of freedom and P value noted<br>*Give P values as exact values whenever suitable.*
☒	☐	For Bayesian analysis, information on the choice of priors and Markov chain Monte Carlo settings
☒	☐	For hierarchical and complex designs, identification of the appropriate level for tests and full reporting of outcomes
☒	☐	Estimates of effect sizes (e.g. Cohen's *d*, Pearson's *r*), indicating how they were calculated

Our web collection on statistics for biologists contains articles on many of the points above.

Software and code

Policy information about availability of computer code

Data collection	All microscopy images were collected on a NIS Elements AR software (Nikon), Aperio ScanScope AT2 (Leica Biosystem) or LSM software (Zeiss). Western blotting data was collected by ImageLab v6.0 (Bio-Rad). Realtime PCR was performed by QuantStudio 6 and 7 Flex Real-Time PCR System. Graph analysis : Graphpad Prism v7, v8 and v9. Cloning : SnapGene software v4.3.4. The processing of scRNA-seq data was primarily carried out using the Seurat package (version v3.1.2) in the R environment (version v3.6.0). Initial data filtering, normalization, scaling, and integration were performed using Seurat and scTransform. Clustering was executed using the Louvain-Jaccard method, and unique marker genes for each cluster were identified using Presto. Differential expression analyses were conducted using Seurat's FindMarkers function, and pathway enrichment analyses were done with gProfiler.
Data analysis	Statistical analyses for cell biological experiments were performed with GraphPad Prism (version 7, 8 and 9) as described in the Method. Software versions: BowTie2, version 2.3.4.1; Samtools, version 1.6.0; BedTools, version 2.26.0; Python, ≥v3.5; Ginkgo; Fiji ImageJ, version 1.53c; SnapGene Software, version 4.3.4; ZEN 2011 microscope software; NIS Elements AR software, version 4.50.

For manuscripts utilizing custom algorithms or software that are central to the research but not yet described in published literature, software must be made available to editors and reviewers. We strongly encourage code deposition in a community repository (e.g. GitHub). See the Nature Portfolio guidelines for submitting code & software for further information.

Data

Policy information about availability of data

All manuscripts must include a data availability statement. This statement should provide the following information, where applicable:

- Accession codes, unique identifiers, or web links for publicly available datasets
- A description of any restrictions on data availability
- For clinical datasets or third party data, please ensure that the statement adheres to our policy

In vivo CRISPR screen sequencing data and Single cell RNA sequencing data are deposited in the NCBI Gene Expression Omnibus. BioSample accession Number : SAMN36935033, SAMN36935034, NCBI BioProject URL : http://www.ncbi.nlm.nih.gov/bioproject/1004263.

Research involving human participants, their data, or biological material

Policy information about studies with human participants or human data. See also policy information about sex, gender (identity/presentation), and sexual orientation and race, ethnicity and racism.

Reporting on sex and gender	N/A
Reporting on race, ethnicity, or other socially relevant groupings	N/A
Population characteristics	N/A
Recruitment	N/A
Ethics oversight	N/A

Note that full information on the approval of the study protocol must also be provided in the manuscript.

Field-specific reporting

Please select the one below that is the best fit for your research. If you are not sure, read the appropriate sections before making your selection.

☒ Life sciences ☐ Behavioural & social sciences ☐ Ecological, evolutionary & environmental sciences

For a reference copy of the document with all sections, see nature.com/documents/nr-reporting-summary-flat.pdf

Life sciences study design

All studies must disclose on these points even when the disclosure is negative.

Sample size	Murine tumorigenesis studies utilized publicly available sample size estimation calculators to attain at least 80% power to detect a 30% reduction in tumor latency using a 2-tailed log rank test. Sample sizes for in vitro experiments were determined empirically from previous experimental experience with similar assays, and/or from sizes generally employed in the field. Specific sample sizes and the count of independent experiments conducted for each study can be found in the figures, their accompanying legends, or within the methods section.
Data exclusions	No data were excluded from analysis or reporting
Replication	All experiments were conducted independently as described in the figure legends or were biologically replicated at least 2-3 times. All p-values were derived from measurements obtained in experiments performed independently at least three times. Representative images or numerically similar results were used, which were replicated and obtained from at least more than two independent biological experiments.
Randomization	The quantification of Immunocytochemistry (ICC) data was obtained from random regions on the slide. For experiments not mentioned here, allocation was not random because data were compared or collected under identical conditions where randomness was not required.
Blinding	For practically feasible (ex_Extended Figure 6b), samples were blinded during analysis. However, the majority of experiments were not conducted under blinded conditions. Most of the data were obtained from at least 2 to 3 biologically independent experiments, were replicated by different investigators, or were validated to produce the same results through different experimental methods.

Reporting for specific materials, systems and methods

We require information from authors about some types of materials, experimental systems and methods used in many studies. Here, indicate whether each material, system or method listed is relevant to your study. If you are not sure if a list item applies to your research, read the appropriate section before selecting a response.

Materials & experimental systems

n/a	Involved in the study
☐	☒ Antibodies
☐	☒ Eukaryotic cell lines
☒	☐ Palaeontology and archaeology
☐	☒ Animals and other organisms
☒	☐ Clinical data
☒	☐ Dual use research of concern
☒	☐ Plants

Methods

n/a	Involved in the study
☒	☐ ChIP-seq
☒	☐ Flow cytometry
☒	☐ MRI-based neuroimaging

Antibodies

Antibodies used

Antibody# Species# Company Cat# Application Dilution
Mre11, Rabbit monoclonal, Novus Biologicals, BN100-142, W.B 1:1,000
β-Actin (clone AC-15), Mouse, Sigma-Aldrich, A1978-100UL, WB 1:10,000
cGAS (D3O8O), Rabbit monoclonal, Cell Signaling Technology, 31659S, Tumor IF 1:4,000, W.B 1,000, ICC 1:500
cGAS (D1D3G), Rabbit monoclonal, Cell Signaling Technology, 15102S, W.B 1:,000, ICC 1:500
α Tubulin, Mouse monoclonal (B-7), Santa Cruz Biotechnology, SC-5286, W.B 1:1000
Phospho-Histone H2A.X (Ser139)(20E3), Rabbit monoclonal, Cell Signaling Technology, 9718S, Tumor IF 1:3,000
STING/TMEM173, Rabbit polyclonal, Novus Biologicals, NBP2-24683, W.B 1:1,000
IRF3 (Ser386)[EPR2346], Rabbit monoclonal, Abcam, ab76493, W.B 1,000
STING (Ser366)(D7C3S), Rabbit monoclonal, Cell Signaling Technology, 19781, W.B 1:1,000
TBK1/NAK (Ser172)(D52C2) , Rabbit monoclonal, Cell Signaling technology, 5483, W.B 1:1,000
GAPDH (G-9), Mouse monoclonal, Santa Cruz Biotechnology, sc-365062, W.B 1:1,000
Rad50, Rabbit polyclonal, Novus Biologicals, NBP2-20054, W.B 1:1,000
NBS1, Mouse monoclonal, Novus Biologicals, NBP2-20554, W.B 1:1,000
Z-DNA antibody, Novous Biologicals NB100-749 ICC 1:500
Horse polyclonal anti-Mouse IgG, HRP-linked antibody, Cell signaling Technology, 7076S, W.B 1:5000
Goat polyclonal anti-Rabbit IgG, HRP linked antibody, Cell signaling Technology, 7074S, W.B 1:5000
Goat polyclonal anti-Hamster IgG, HRP-linked antibody, Thermo Fisher Scientific, PA1-29626, W.B 1:5000
HaloTag® protein antibody, Mouse monoclonal, Promega, G9211, W.B 1,000, ICC 1:500
MLKL (S345)[EPR9515(2)], Rabbit monoclonal, Abcam, ab196436, W.B 1:1,000, ICC 1:500
MLKL antibody, Mouse monoclonal, Proteintech, 66675-1-Ig, W.B 1:1000
ZBP1(Zippy-1), Mouse monoclonal, AdipoGen, AG-20B-0010, W.B 1:1,000
ATM(D2E2), Rabbit monoclonal, Cell Signaling Technology, 2873S, W.B 1:1000
Goat anti-Rabbit IgG (H+L) Secondary Antibody, Alexa Fluor 488, Thermo Fisher Scientific, A11034, ICC : 1:500
Goat anti Rabbit IgG (H+L) Secondary Antibody, Alexa Fluor 594, Thermo Fisher Scientific, A11037, ICC : 1:500
F(ab')2-Goat anti-Rabbit IgG (H+L) Cross-Adsorbed Secondary Antibody, Alexa Fluor 633, Thermo Fisher Scientific, A11072, ICC : 1:500
Cy™3 AffiniPure Donkey Anti-Mouse IgG (H+L), Jackson ImmunoResearch, 715-165-151, ICC : 1:500
Cy™5 AffiniPure Goat Anti-Rabbit IgG (H+L), Jackson ImmunoResearch, 111-175-144, ICC : 1:500
Streptavidin, Alexa Fluor™ 488 Conjugate, Thermo Fisher Scientific, S32354, ICC : 1:500
Z-DNA, Sheep Polyclonal, Novous Biologicals, NB100-749, ICC 1:500
Donkey anti-Sheep IgG (H+L) Cross-Adsorbed Secondary Antibody, Alexa Fluor™ 594 Invitrogen™, Invitrogen, A-11016, ICC 1:500

Validation

Appropriate positive and negative controls were included in the experimental design to confirm the antibodies were specific. Critical antibodies were validated by depletion or knockout of target genes using RNA interference or CRISPR/Cas9 editing (lentivirus), respectively.

Eukaryotic cell lines

Policy information about cell lines and Sex and Gender in Research

Cell line source(s)

SF9 (Thermo Fisher Scientific, 11496015), WT MEFs (Gift from John Petrini, Ph.D), ATLD/ATLD MEFs (Gift from John Petrini, Ph.D), BJ-5ta (ATCC, CRL-4001), MDA-MB-231 cell lines (ATCC, CRM-HTB-26), Lucia ISG Cells (Invivogen, rawl-isg), HEK293T/17 (ATCC® CRL-11268), primary murine mammary epithelial cell lines (pMMECs) from female mice. As cited in method.

Authentication

We used DNA fingerprint analysis and PCR genotyping for authentication (MEFs, BJ-5ta, MDA-MB-231 cell lines). For cell lines not mentioned in this field such as the sf9, Lucia ISG Cells and pMMEC cell lines were not authenticated; pMMEC was directly isolated from from female mice and used without additional authentication, and sf9 and Lucia ISG Cells were delivered from the company and used immediately.

Mycoplasma contamination

All cells are routinely tested for and found to be mycoplasma free as described in the Methods.

Commonly misidentified lines
(See ICLAC register)

We never use misidentified cell lines.

Animals and other research organisms

Policy information about studies involving animals; ARRIVE guidelines recommended for reporting animal research, and Sex and Gender in Research

Laboratory animals	Mus musculus; FVB.129P2-Trp53tm1Brn/Nci, C57BL/6N-Gt(ROSA)26Sortm13(CAG-MYC,-CD2*)Rsky/J, B6;129-Gt(ROSA)26Sortm1(CAG-cas9*,-EGFP)Fezh/J, NOD.129S7(B6)-Rag1tm1Mom/J. We have detailed the information on animals and housing conditions for mice in the Materials and Methods section.
Wild animals	Wild animals were not used in this study.
Reporting on sex	We exclusively utilized female mice for our study/experiments and outlined their respective ages for each experiment in the Methods section.
Field-collected samples	Samples collected in the field were not used in this study.
Ethics oversight	The UNC Institutional Animal Care and Use Committee (IACUC) approved guidance on our animal study protocol. IACUC ID : 22-163.0. The mice were euthanized in a humane manner in accordance with the guidelines set by IACUC when they reached a predetermined experimental endpoint.

Note that full information on the approval of the study protocol must also be provided in the manuscript.
